# Growth Responses of Preterm Pigs Fed Formulas with Different Protein Levels and Supplemented with Leucine or β-Hydroxyl β-Methylbutyrate

**DOI:** 10.3390/nu10050636

**Published:** 2018-05-18

**Authors:** Randal K. Buddington, Scott C. Howard, Harold W. Lee, Karyl K. Buddington

**Affiliations:** 1 School of Health Studies, University of Memphis, Memphis, TN 38152, USA; hwlee@memphis.edu; 2 College of Nursing, University of Tennessee Health Sciences Center, Memphis, TN 38163, USA; showard5@uthsc.edu; 3 Biological Sciences, University of Memphis, Memphis, TN 38152, USA; kbudding@memphis.edu

**Keywords:** prematurity, preterm infant, growth, leucine, β-hydroxy β-methylbutyrate, formula

## Abstract

Growth after preterm birth is an important determinant of long-term outcomes. Yet, many preterm infants suffer ex utero growth retardation. We evaluated effects of leucine and the metabolite, β-hydroxy β-methylbutyrate (HMB) on growth of preterm pigs, a previously-validated translational model for preterm infants. After 48 h of parenteral nutrition preterm pigs were fed for 6 to 7 days isocaloric formulas with different levels of protein (50 or 100 g/L) with leucine (10 g/L, 76 mM) or HMB (at 1.1 g/L, 4 mM) added to stimulate protein synthesis or with alanine (6.8 g/L; 76 mM) as the control. Rates of growth of pigs fed the low protein formula with alanine (3.4 ± 0.2% gain per day) or leucine (3.7 ± 0.2) exceeded that of pigs fed the high protein formula (2.8 ± 0.2, *p* = 0.02 for comparison with both low protein formulas; *p* = 0.01 compared with low protein + leucine). Supplementing the high protein formula with leucine or HMB did not increase growth relative to alanine (2.72 ± 0.20, 2.74 ± 0.27, and 2.52 ± 0.20, respectively). Small pigs (<700 g birth weight) grew slower during parenteral nutrition and had a more pronounced response to leucine. Females fed the high protein formulas grew faster than males, and particularly for small pigs (*p* < 0.05). Blood urea nitrogen values were lower for pigs fed the low versus the high protein formulas (*p* < 0.05). Leucine and HMB improved growth of preterm pigs fed low, but not high protein formulas, even after controlling for birth weight and sex, which independently correlated with growth rates. They offer an option to improve growth without increasing the amino acid load, with its attendant metabolic disadvantages.

## 1. Introduction

Preterm birth is the leading cause of infant death and developmental disabilities [1] and the risk of poor outcomes is increased further with extrauterine growth retardation (EUGR) [2,3]. A goal of preterm nutrition support is to match the rapid growth of fetuses between 22 and 40 weeks of gestation and protein accretion of 2 to 2.5 g/kg-day [4,5,6] and thereby improve outcomes [7]. Preterm infants are provided 2.5 to 3.5 g of amino acids/kg-day while reliant on parenteral nutrition (PN) [8] and 3 to 4 g of dietary protein/kg-day when fed formulas [9]. Because the protein content of breast milk is not considered adequate [9], fortifiers are routinely used to increase protein content up to 4 g/kg-day. However, the high amounts of protein can compromise growth of preterm infants [10,11], increase the risk of obesity later in life [12], and overload the immature abilities of the hepatic and renal systems to handle excessive nitrogenous wastes, leading to acidosis, elevated blood urea nitrogen (BUN), and hypernatreimic dehydration [10]. Achieving acceptable growth without providing excessive amino acid/protein is desirable.

Leucine or the associated metabolite, β-hydroxy β-methylbutyrate (HMB) are known to activate the mTORC1 signaling pathway and induce skeletal muscle protein synthesis and attenuate protein degradation independent of insulin [13,14]. The mTORC1 signaling system is expressed by single-celled and early stage embryos [15] and is important for development [16]. Corresponding with this, neonatal term pigs respond to leucine and HMB with increased skeletal muscle protein synthesis [14,17], even when energy and protein are restricted [18]. Furthermore, pigs born small for gestational age also exhibit increased muscle protein synthesis in response to supplemental leucine and HMB [19,20]. More effective nutrition support strategies that promote growth are needed for preterm infants already at risk of extrauterine growth retardation, and associated poor outcomes. This led us to hypothesize that preterm pigs, a recognized translational model for preterm infants, [21] would also respond with improved growth when fed formulas supplemented with leucine or HMB to activate the mTORC1 pathway. We initially determined if the responses of neonatal term pigs to leucine [18] were shared by preterm pigs. This was done by measuring growth of preterm pigs that were fed two formulas with protein comparable to mature sow milk, with and without leucine, or fed a formula with double the protein. This addressed the question of whether growth could be improved by supplemental leucine without additional protein. After observing lower growth by pigs fed the high protein formula, a second experiment evaluated if supplementing the high protein formula with leucine or HMB would improve growth.

## 2. Materials and Methods

### 2.1. Animal Care

The University of Memphis Institutional Animal Care and Use Committee approved all aspects of the project involving the care and use of pigs (approval number 0674). Preterm pigs were delivered by caesarian section at 92% of term (day 105 of 115-day gestation). Within 3 h after delivery, an umbilical artery catheter (UAC) was placed, a feeding tube was inserted via a small incision in the cheek, and maternal serum (5–6 mL) was administered via the UAC as a source of passive immunity. All of the pigs received an injection (intramuscular) of iron dextran (1 mL; 200 mg of dextran suspension of elemental iron).

The pigs in each litter were separated into three relative sizes (small, intermediate, and large) without consideration of sex. Pigs in each size category were randomly distributed to control and treatment groups such that each group had comparable size distributions to account for the influence of birthweight on postnatal growth [22].

### 2.2. Nutrition Support

The same placebo controlled study design was used for two experiments that evaluated growth responses to different levels of protein and supplemental leucine or HMB. Parenteral nutrition (PN) only was provided for the first 45 to 48 h at a rate of 8 mL/kg-h using an all-in-one solution with (per L) 989 Kcal as glucose (116.3 g), amino acids (60.5 g; Travasol), and fat (31.3 g; Intralipid), with electrolytes, minerals, and vitamins (Pediatric Infuvite and MTE-4). The pigs were transferred abruptly to only full enteral nutrition (EN) for 6 or 7 days using bolus feeding of 15 mL/kg of experimental formulas every 3 h, with volumes adjusted daily based on that day’s weight. The volume of food fed per day (120 mL/kg) is less than what is consumed by term pigs, but does not induce intolerance, and is relevant to the lower volumes fed to preterm compared with term infants.

#### 2.2.1. Experiment 1

A total of 72 pigs were delivered from five sows and 62 were transitioned from PN to being fed three experimental formulas with two levels of protein (Table 1) and lactose as the sole source of carbohydrate to reduce the risk of necrotizing enterocolitis [23]. Similar to a previous study with neonatal term pigs [14], two formulas with 50 g of protein/L, slightly lower than mature sow milk [24], were supplemented with 76 mM of either leucine (10 g/L; LP+Leu) or alanine (6.8 g/L; LP+Ala) as a placebo.

A high protein formula (HP) provided 100 g of protein/L, which is about two-fold higher than mature sow milk, but less than the 150–170 g/L present in sow colostrum [25,26]. The higher protein level compared with mature sow milk was included to mimic the elevated protein levels fed to preterm infants compared with breast milk. The LP+Leu and HP formulas had comparable leucine content, but were not isonitrogenous. Medium chain triglyceride oil was added to the two low protein formulas to be isocaloric with the high protein formula, with all formulas having 1027 Kcal/L.

#### 2.2.2. Experiment 2 

Another 83 pigs were delivered from six sows and 75 were transitioned from PN and used to determine if the low growth response to the high protein formula could be increased by the supplement of 76 mM leucine (HP+Leu; 10 g/L) or by 1.1 g/L (4 mM) Ca-HMB monohydrate (HP+HMB). The high protein formula with alanine (HP+Ala; 76 mM) was used as the placebo control (Table 2). The 1.1 g/L HMB is ~6 times higher than what had been fed to newborn term pigs [17,19]. A higher HMB formula (11.2 g/L) fed to 7 pigs in the first two litters did not improve growth (2.44 ± 0.12 vs. HP+HMB = 2.55 ± 0.11; *p* = 0.24) and was not included in the remaining litters. Calcium levels were equilibrated by adding calcium acetate monohydrate to the HP+Ala and HP+Leu formulas.

### 2.3. Assessment of Responses

Body weights were recorded daily after the start of EN. Hematology was assessed at the end of PN and at necropsy (Abaxis VM2 hematology platform) and clinical chemistries were performed during PN and until conclusion of EN (Abaxis Vetscan; Comprehensive Diagnostic Panel, Union City, CA, USA). Body composition was not determined.

### 2.4. Statistics

Values in figures and tables are means and standard errors. Analysis of covariance was used to evaluate the potential confounders of litter and birth weight. The final multiple variable model associated treatment and measured outcomes, adjusting for birthweight, litter, and day of life. Sex was included as a variable after initial evaluations revealed a potential influence. Unpaired *t*-tests were used for selected comparisons. All analyses were completed using SAS v.9.1.3 (SAS Institute, Cary, NC, USA) with *p* < 0.05 accepted as the critical level of significance.

## 3. Results

### 3.1. General Observations and Results

The early deaths that occurred immediately after delivery and during the 48 h of PN of Experiment 1 (*n* = 10; 14%) and Experiment 2 (*n* = 8; 10%) are similar to the higher mortality of newborn preterm infants. Deaths during the 6 to 7 days of formula feeding (7/62 and 7/75 for Experiments 1 and 2) were evenly distributed across groups and necropsies did not reveal the cause of death for most of the pigs. Only pigs that completed the 48 h of PN and survived the entire 6 or 7 days of EN were included in the analysis. The immediate change from PN to only EN experienced by the pigs, contrasts with the gradual transition used for preterm infants. There were no signs of formula intolerance or poor digestibility of any of the formulas (bloating, vomiting) and none of the pigs had lesions consistent with necrotizing enterocolitis, including those that died prior to scheduled necropsy.

During the 48 h of PN growth of the smallest pigs (<700 g at birth; 9.0 ± 1.1% of birthweight gained) was lower compared with pigs weighing >700 g at birth (13.3% ± 0.4%; *p* < 0.05). Weight gain during PN for the largest pigs (>1100 g at birth; 12.8 ± 0.6% birth weight gained) was not different relative to pigs weighing between 700 and 1100 g a birth (13.6 ± 0.5%). Sex did not influence growth during PN with similar weight gains for male and female pigs with birth weights <700 g (*p* = 0.24) or >700 g (*p* = 0.56).

### 3.2. Growth during EN

#### 3.2.1. Experiment 1

Birthweights for the 55 pigs that completed the study (76%) ranged from 247 to 1847 g. The litter sizes ranged from 5 to 23 with larger litters having on average smaller pigs, contributing to litter as a confounding variable. There were 27 females and 28 males. Although birthweights did not differ among treatments (Table 3), they were slightly lower for HP pigs by the assignment of the heavier pigs from the litter with only 5 pigs to the two low protein groups. The average numbers of days of EN were comparable among the three treatment groups (6.4 days, 6.4 days, and 6.5 days). Pigs that died during the period of EN before scheduled necropsy were evenly distributed among treatment groups (2 HP, 2 LP+Leu, 1 LP+Ala).

**Figure 1:**
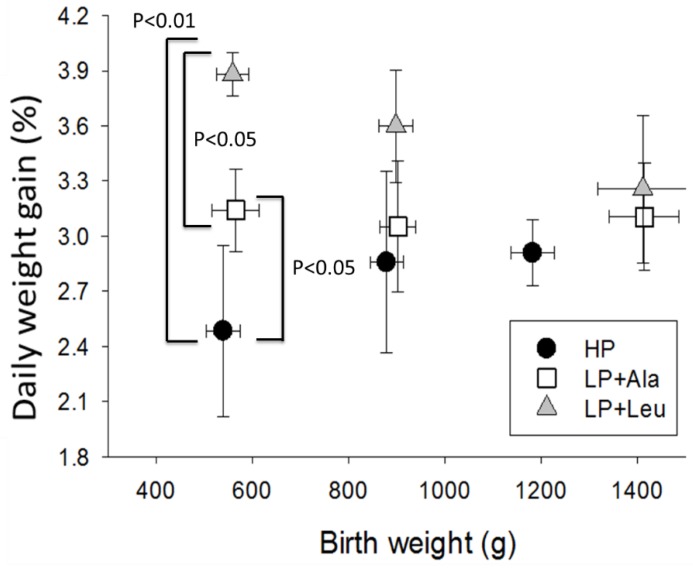
Growth rates as percentage gains per day for Experiment 1 pigs with different birth weights and fed the high protein formula (HP) and the low protein formula supplemented with alanine (LP+Ala) or leucine (LP+Leu).

Weight gains during EN varied widely, even within individual treatments, with a significant effect of litter (*p* < 0.05) and birth weight (*p* < 0.01). Contrary to expectations, the growth of HP pigs (2.8 ± 0.2%/day) was lower compared to pigs fed the two low protein formulas (LP+Ala = 3.4 ± 0.2 and LP+Leu = 3.7 ± 0.2; *p* < 0.01 for pooled data and *p* < 0.01 for LP+Leu). For the smallest pigs (<700 g), the LP+Leu group grew significantly more than the LP+Ala group (Figure 1; *p* = 0.05).

#### 3.2.2. Experiment 2

Birth weights ranged from 565 to 1443 g for the 68 pigs that completed the study (91%) and did not differ among the four treatments (Table 3). Days of EN averaged 6.6 ± 0.1, 6.3 ± 0.2, and 6.6 + 0.1 for HP+Ala, HP+Leu, and HP+HMB, respectively.

**Figure 2:**
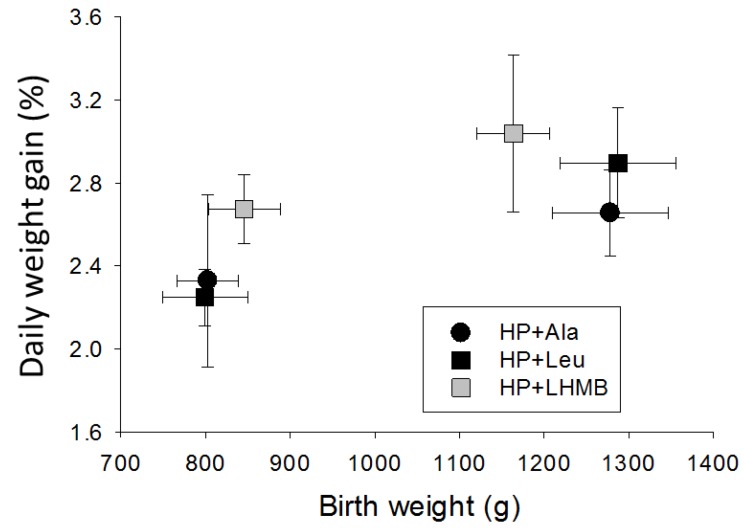
Growth rates as percentage gain per day for Experiment 2 pigs fed the high protein formula supplemented with alanine (HP+Ala), leucine (HP+Leu), or the lower dose of β-hydroxyl β-methylbutyrate (LHMB).

Growth (%/day) varied among individuals within treatments and litters, with a significant influence of birth weight (*p* < 0.01). The HP+Leu and HP+HMB formulas did not appreciably increase the rate of weight gain compared to HP+Ala, for smaller or larger pigs (Figure 2). Growth of HP+Ala pigs (2.52 ± 0.19) was comparable to Experiment 1 HP pigs (2.74 ± 0.28), but was significantly lower than the LP+Leu and LP+Ala pigs in Experiment 1 (pooled data; *p* < 0.05).

### 3.3. Hematology and Blood Chemistries

White blood cell counts increased in all pigs during EN, and particularly lymphocytes, and platelets. Although red blood cell densities did not decrease, because mean red blood cell volume and hemoglobin content declined, hematocrit and total hemoglobin were lower at conclusion of the feeding period (Supplemental Table S1). During the 48 h of parenteral nutrition glucose, total protein, alanine aminotransferase, total bilirubin, BUN increased, whereas phosphate and creatinine declined (Supplemental Table S2).

#### 3.3.1. Experiment 1

The LP+Ala and LP+Leu pigs had more pronounced increases in platelet counts compared with HP pigs (*p* < 0.05).

BUN decreased in all groups after the start of EN (Supplemental Table S2), but more so for pigs fed the low protein formulas (*p* values < 0.05), which did not differ, with some samples below the limit of detection (<2 mg/dL). Glucose and calcium decreased after the start of feeding in all groups and remained lower, whereas total protein increased. HP pigs consistently had lower amylase and glucose values, but higher total protein compared to the similar values for pigs fed the two low protein formulas. LP+Leu pigs had higher alkaline phosphatase than HP and LP+Ala pigs.

#### 3.3.2. Experiment 2

The HP+HMB pigs had higher red blood cell counts and hematocrit, with more hemoglobin, due partly to slightly larger erythrocytes with a higher hemoglobin content. Platelet counts did not differ among the groups and were similar to those of HP pigs in experiment 1.

The only difference in blood chemistries (Supplemental Table S3) was the higher BUN for the HP+HMB pigs, with values for the HP+Ala and HP+Leu pigs similar and comparable to those of the HP pigs in experiment 1.

## 4. Discussion

The aggressive nutrition support provided to the preterm pigs prevented the EUGR common among preterm infants [3] and the weight gains of 5 to 6% per day during the 48 h of PN exceed the 4 to 5% gained per day by fetal pigs in late gestation [27,28]. The slower growth during the period of EN (3.0 ± 0.1%/day; pooled data for all pigs >1000 g at necropsy) corresponded with the volume of formula fed providing less fluid, energy, and protein than during PN. Moreover, to avoid intolerance the amount fed was <50% of milk voluntarily consumed by newborn term pigs [29]. As a result, growth was less than the ~10% gain per day typical of suckling term pigs during the first 14 days after birth [30], but was similar to and even higher than many other studies using preterm pigs fed formula.

### 4.1. Growth Responses to Different Levels of Protein

Suckling term pigs grow better when fed a high protein formula [31], with similar evidence for preterm infants [9]. This led to the a priori expectation the high protein formulas would elicit better growth in preterm pigs as well. At the rate fed, the high protein formulas provided 12 g of protein/kg-day. Although this exceeds the protein accretion rate of 4.63 g/kg-day by fetal pigs [27], it would be considered marginal for 5 to 7 day old suckling pigs born at term [31,32], with the two low protein formulas providing 50% less.

The higher growth of pigs fed the two low protein formulas (3.30 ± 0.14%/day vs. 2.70 ± 0.13; *p* < 0.001; pooled data for the two low and four high protein formulas respectively), despite similar birth weights (1025 ± 52 vs. 1041 ± 44 g; *p* = 0.78) was unexpected. The MCT oil added to keep the low protein formulas isocaloric may have increased energy availability. The immature lipid digestion of preterm pigs [33] may limit available energy from the lard that is the principal component of the dry fat added to the formulas. However, symptoms of steatorrhea were not evident. MCT oil is a readily available source of energy [34] and may provide a protein sparing effect, consistent with the lower BUN of the low protein pigs. The higher BUN and lower growth associated with the high protein formulas indicates amino acids were being deaminated, rather than directed into protein synthesis, which may have contributed to the reduced growth. There is a need to understand better the relationship between protein and energy intakes and growth and body composition [9].

### 4.2. Responses to Leucine and HMB

The enhanced growth response to leucine with the low protein formula (LP-Ala = 3.22 ±0.16 vs. 3.62 ±0.10; *p* = 0.04) is consistent with increased growth responses of suckling pigs to leucine [30,35], activation of the mTORC1 pathway [18], and the potential to elicit anabolic responses. The increased growth of LP+Leu compared with HP pigs, despite the lower protein intake, contrasts with the diminished response to leucine of suckling pigs fed formulas with restricted protein and energy [18].

The surprisingly lower growth of the HP pigs led us to determine in Experiment 2 if adding leucine or HMB would improve growth. The insignificant growth responses to leucine and HMB compared with HP+Ala pigs (2.78 ± 0.15 pooled data for HP+Leu and HP+HMB pigs vs. 2.52 ± 0.20 for the HP-ALA pigs; *p* = 0.27) and the growth of HP pigs in Experiment 1 indicated little growth benefit by adding leucine or HMB to the higher protein formula. Although the mTORC1 signaling pathway is expressed early in development and was not evaluated in the present study, the findings indicate there is a need to determine if and how different protein levels influence the mTORC1 signaling pathway of preterm pigs and the responses to leucine and HMB.

At the rate fed, the HP+HMB formula provided a dose of 480 μmol/kg-day. This is 6 to 7-fold higher than the highest levels fed to newborn term pigs that increased fractional rates of protein synthesis in several tissues (80 μmol/kg-day [36]) and the 70 μmol/kg-day that increased growth of IUGR term pigs [19]. Although not measured in the present study, plasma HMB concentrations are reported as ~1/1000 of the enteral dose [36]. Although speculative, the HP+HMB pigs would potentially have plasma concentrations of ~400 nmol. This is comparable to the concentrations after an acute oral dose of 40 μmol/kg-day that increased fractional rates of protein synthesis in skeletal muscle [17]. The HP+HHMB provided 4800 μmol/kg-day with plasma concentrations likely exceeding 4000 nmol. Although there no overt signs of toxicity, growth was not increased.

### 4.3. The Influences of Birth Weight and Sex 

Being born both premature and small for gestational age compromises postnatal growth of infants [37] and further increases the risk of adverse outcomes [38,39]. Similarly, fetal and postnatal growth rates are related for pigs [22] and intrauterine growth restricted pigs grow slower than normal birth weight littermates [40,41]. Typically, about 10% of newborn term pigs weigh <800 g at term due to intrauterine growth restriction [42] leading us to consider the 22 preterm pigs <700 g at 105 days of gestation (~15% of the 147 pigs delivered) to be small for gestational age (SGA). These smaller pigs had lower growth compared to larger pigs during the 48 h of PN and during EN when the high protein formulas were fed (*p* < 0.05). For unexplained reasons, the larger pigs did not experience significantly increased growth when fed the low protein formulas (*p* = 0.67), contrary to their smaller counterparts. There is a need to determine if the influence of birth weight on growth responses of preterm pigs to different protein intakes also applies to preterm infants.

The greater growth of the SGA pigs in Experiment 1 (Section 3.2.1) fed the low protein formula with leucine is consistent with more pronounced stimulation of mTORC1 by leucine and HMB of term pigs that are small for gestational age [19,20]. Although this study was limited to 6–7 days, the 30% higher rate of growth for the smaller compared to the larger pigs when fed the LP+Leu formula is novel and indicates leucine has the capacity to elicit compensatory growth. However, supplemental leucine and HMB did not improve growth of the SGA pigs when fed the high protein formula (2.49 ± 0.15 pooled data for HP+Leu and HP+HMB vs. 2.34 ± 0.50 for HP+Ala; *p* = 0.76).

**Figure 3:**
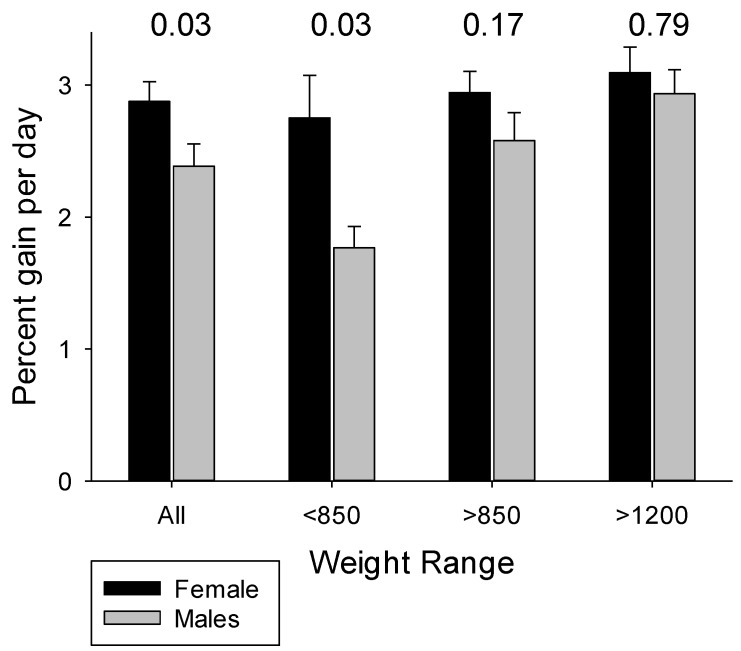
Birth weight and sex influence rate of weight gain by preterm pigs fed the high protein formulas fed in Experiments 1 and 2 (pooled data for HP, HP+Ala, HP+Leu, HP+HMB). *p*-values above paired data are for comparison of females versus males.

Pooled data for the pigs in Experiments 1 and 2 fed the four high protein formulas revealed higher growth for female pigs (Figure 3), despite similar birth weights for males (1004 ± 53 g for females vs. 1055 ± 60 g for males; *p* = 0.71), with the differences especially evident for the smaller pigs. These findings are consistent with the lower morbidity and mortality and better outcomes for female preterm infants, and especially SGA infants [43,44]. Interestingly, growth was similar for female and male pigs when fed the low protein formulas (*p*’s > 0.50).

### 4.4. Hematology and Clinical Chemistries

Compared with values for preterm infants [45,46], preterm pigs have smaller red blood cells and despite higher densities, have a lower hematocrit and less hemoglobin per mL. Newborn preterm pigs and human infants have similar platelet counts and both species have postnatal increases. The white blood cell counts at birth are lower for preterm pigs compared to preterm infants. They increase rapidly during the first week after delivery and remain dominated by lymphocytes. It is unknown if the contrasting postnatal patterns of change for white blood cell counts are related to species, stage of development, or aspects of care and handling. The lower platelet counts among pigs fed the high protein formula is unexplained.

The present study contributes needed reference data for clinical chemistries of preterm pigs (Supplemental Tables S2 and S3). The hyperglycemia of the preterm pigs during PN mimics preterm infants [47], and may reflect a combination of stress responses to birth and immature glucoregulation, suggesting the 22 g/kg-day of glucose in the PN solution may be excessive. The lower blood glucose levels during EN for all treatments may be considered as slightly hypoglycemic. At conclusion of EN, blood chemistries for the pigs fed the high protein formulas were similar to those of preterm pigs fed for 6 to 7 days a lactose-based formula with 83 g of protein per L [23].

The BUN values measured during PN are comparable to preterm infants receiving primarily parenteral nutrition during the first week after delivery [48]. The low and sometimes undetectable BUN levels for preterm pigs fed the low protein formulas are comparable to those of term pigs fed low protein formulas [49] and preterm infants fed a low protein formula that still promotes growth [50]. The low BUN values suggest that the majority of the protein was partitioned into accretion of lean body mass, with little catabolism of dietary or tissue protein along with lower energetic costs for nitrogen disposal. However, BUN values <4 mg/dL are indicative of inadequate protein intake [51]. The higher BUN values for pigs fed the high protein formulas would not be considered excessive (i.e., >50 mg/dL). It is unknown why HP+HMB pigs had higher BUN, despite slightly increased weight gain and PER relative to HP+Ala and HP+Leu pigs, suggesting a greater proportion of dietary protein was converted into tissue.

## 5. Conclusions

The preterm pig is a relevant large animal model for understanding the consequences of being born early, small, male or female, and for investigating nutrition support. The ability to improve growth using supplemental leucine or HMB without excessive protein, particularly for SGA pigs, warrants further study, as does the use of MCT oil and other sources of energy for protein sparing. The growth responses of preterm pigs to varying protein intakes with and without supplemental leucine and HMB may differ from those of term pigs because of different post conception ages [52] and because gastrointestinal immaturity limits tolerated dietary loads. It will be important to measure body composition to determine if leucine and HMB increase the proportion of dietary energy and protein directed into accretion of lean body mass rather than adipose tissue. Elucidating the effects of growth promoting ingredients should support improvements in nutritional strategies.

## Figures and Tables

**Table 1:** Macronutrient composition of the high (HP) and low protein formulas supplemented with either alanine (LP+Ala) or leucine (LP+Leu) fed to preterm pigs in Phase 1. Ingredient details are provided as supplementary material.

	HP	LP+Ala	LP+Leu
Ingredient (g/L)			
Protein	100	50	50
Lipid	47.5	47.5	47.5
Medium Chain Triglyceride oil	0	22.5	22.5
Lactose	50	50	50
Alanine	0	6.9 (76 mM)	0
Leucine	0	0	10 (76 mM)
Vitamins and Minerals	5	5	5
LEC/STAR487/MO B	4.4	4.4	4.4

**Table 2:** The experimental formulas used for Experiment 2 to evaluate the responses of preterm pigs to the high protein formula used in Phase 1 (100 g protein per L) supplemented with alanine (HP+Ala), leucine (HP+Leu) or β-hydroxyl β-methylbutyrate (HP+HMB).

	HP+Ala	HP+Leu	HP+HMB
Component	g/L		
Alanine	6.8 (76 mmol)	0	0
Leucine	0	10 (76 mmol)	0
HMB	0	0	1.1 (4 mmol)
Ca-Acetate	0.6 (6.1 mmol)	0.6 (6.1 mmol)	0 (5.5 mmol)

**Table 3:** Birth weights of pigs in Experiments 1 and 2 and fed the low and high protein formulas with and without leucine, HMB, or alanine. The percentages of females are in parentheses.

Experiment 1			
Group	HP	LP+Ala	LP+Leu
Birth Weight (g)	795 ± 80 (47)	1053 ± 73 (41)	997 ± 70 (61)
Experiment 2			
Group	HP+Ala	HP+Leu	HP+HMB
Birth Weight (g)	1147 ± 77 (67)	1122 ± 66 (63)	1054 ± 63 (36)

## Supplementary Materials

The following are available online at http://www.mdpi.com/2072-6643/10/5/636/s1, File S1: Ingredients used to prepare the low and high protein formulas, Table S1: Hematology values for Experiment 1 and 2 pigs, Table S2: Blood chemistries for Experiment 1 pigs, Table S3: Blood chemistries for Experiment 2 pigs.
