# Supplementary material for: Growth Responses of Preterm Pigs Fed Formulas with Different Protein Levels and Supplemented with Leucine or β-Hydroxyl β-Methylbutyrate
Source: Nutrients. 2018 May 18;10(5):636. doi: 10.3390/nu10050636 (PMC5986515; doi:10.3390/nu10050636)
Supplement: Supplementary file 1 [file nutrients-10-00636-s001.zip › Nutrients-293566 Suppl Table 2.docx]

Supplemental Table S2. Blood chemistries for Experiment 1 pigs immediately after birth (day -2), after 24 (-1) and 48 h (0) of parenteral nutrition, and during feeding of the high protein formula (HP) and the two low protein formulas with alanine (LP+Ala) and leucine (LP+Leu) for 6 or 7 days. Values are means and standard errors.

| Day | ALP^1^ (U/L) | ALT (U/L) | AMY (U/L) | TBIL (mg/dL) | BUN (mg/dL) | CA++ (mg/dL) | PHOS (mg/dL) | CR  (mg/dL) | GLU (mg/dL) | Na+ | K+ | TP (g/dL) |
| --- | --- | --- | --- | --- | --- | --- | --- | --- | --- | --- | --- | --- |
| -2 | 904+102 | 11+0 | 659+50 | 0.26+0.01 | 14.8+1.0 | 12.3+0.2 | 7.0 +0.1 | 1.1 +0.1 | 42+3 | 141+1 | 4.1+0.1 | 1.9+0.1 |
| -1 | 625+47 | 14+1 | 536+ 32 | 0.46+0.04 | 21.0+1.3 | 13.0+0.2 | 4.2 +0.3 | 0.6 +0.0 | 290+37 | 137+1 | 6.1+0.2 | 2.2+0.1 |
| 0 | 815+59 | 16+ 1 | 537+ 35 | 0.37+0.02 | 16.7+1.7 | 12.9+0.3 | 5.2 +0.3 | 0.5 +0.0 | 184+26 | 138+1 | 5.7+0.1 | 2.6+0.0 |
| 1 HP | 1042+135 | 16+1 | 552+45 | 0.38+0.02 | 12.0+1.5 | 11.2+0.5 | 5.4+0.2 | 0.5+0.0 | 59+5 | 141+1 | 5.3+0.2 | 2.9+0.0 |
| LP+Ala | 788+127 | 16+2 | 650+43 | 0.28+0.02 | 5.2+1.6 | 11.4+0.7 | 5.7+0.4 | 0.5+0.0 | 69+4 | 142+1 | 5.3+0.2 | 2.9+0.1 |
| LP+Leu | 1291+270 | 22+6 | 604+84 | 0.33+0.06 | 8.3+4.2 | 11.9+0.4 | 6.0+0.5 | 0.4+0.1 | 77+8 | 139+3 | 5.5+0.1 | 2.7+0.2 |
| 2 HP | 973+125 | 17+1 | 554+56 | 0.32+0.02 | 16.9+1.8 | 11.2+0.2 | 5.9+0.2 | 0.4+0.1 | 62+5 | 139+1 | 5.0+0.1 | 3.3+0.0 |
| LP+Ala | 841+241 | 18+2 | 707+61 | 0.30+0.00 | 1.0+0.0 | 11.7+0.3 | 5.3+0.3 | 0.5+0.0 | 77+9 | 142+1 | 5.6+0.2 | 3.1+0.1 |
| LP+Leu | 1048+143 | 17+1 | 671+57 | 0.28+0.02 | 1.5+0.5 | 11.2+0.3 | 5.1+0.1 | 0.5+0.0 | 73+4 | 140+1 | 5.7+0.2 | 2.9+0.1 |
| 3 HP | 814+88 | 16+1 | 536+54 | 0.29+0.01 | 21.2+2.1 | 10.9+0.2 | 6.4+0.2 | 0.4+0.0 | 61+3 | 138+1 | 4.9+0.1 | 3.6+0.1 |
| LP+Ala | 750+115 | 17+1 | 735+50 | 0.25+0.02 | 1.3+0.4 | 11.1+0.5 | 5.6+0.3 | 0.5+0.0 | 76+4 | 140+1 | 5.6+0.3 | 3.2+0.1 |
| LP+Leu | 1022+141 | 17+1 | 697+59 | 0.28+0.02 | 1.6+0.5 | 11.5+0.1 | 5.1+0.1 | 0.5+0.0 | 75+4 | 140+1 | 5.3+0.1 | 3.1+0.0 |
| 4 HP | 638+48 | 16+1 | 529+59 | 0.26+0.02 | 25.4+1.6 | 10.2+0.4 | 6.4+0.1 | 0.4+0.0 | 53+4 | 139+1 | 5.3+0.2 | 3.8+0.1 |
| LP+Ala | 647+89 | 16+2 | 721+53 | 0.30+0.03 | 1.3+0.2 | 11.3+0.2 | 5.7+0.2 | 0.4+0.0 | 64+5 | 139+1 | 5.3+0.1 | 3.4+0.1 |
| LP+Leu | 874+124 | 16+1 | 672+57 | 0.29+0.01 | 2.3+0.5 | 10.9+0.1 | 5.4+0.2 | 0.4+0.0 | 75+7 | 138+2 | 5.2+0.1 | 3.3+0.1 |
| 5 HP | 571+43 | 14+1 | 518+60 | 0.38+0.12 | 30.0+1.7 | 10.7+0.3 | 6.2+0.2 | 0.4+0.0 | 63+4 | 139+1 | 5.2+0.2 | 3.9+0.1 |
| LP+Ala | 605+43 | 16+1 | 661+79 | 0.25+0.02 | 1.3+0.4 | 11.0+0.3 | 5.8+0.2 | 0.5+0.0 | 72+7 | 137+1 | 5.1+0.1 | 3.4+0.1 |
| LP+Leu | 740+120 | 14+1 | 636+60 | 0.28+0.02 | 2.3+0.4 | 11.0+0.2 | 5.8+0.2 | 0.4+0.0 | 67+4 | 139+1 | 5.3+0.2 | 3.4+0.0 |
| 6/7 HP | 525+40 | 15+1 | 539+75 | 0.26+0.03 | 28.3+2.0 | 10.7+0.3 | 6.2+0.1 | 0.4+0.0 | 72+14 | 139+1 | 4.9+0.2 | 4.1+0.1 |
| LP+Ala | 560+28 | 16+1 | 757+67 | 0.25+0.02 | 1.3+0.4 | 10.4+0.2 | 6.1+0.3 | 0.5+0.1 | 93+12 | 138+1 | 4.7+0.2 | 3.5+0.1 |
| LP+Leu | 711+145 | 16+2 | 685+70 | 0.28+0.02 | 2.7+0.5 | 10.8+0.2 | 6.1+0.3 | 0.5+0.1 | 87+13 | 138+1 | 5.2+0.2 | 3.5+0.1 |

^1^ ALP, alkaline phosphatase; ALT, alanine aminotransferase, AMY, amylase; TBIL, total bilirubin; BUN, blood urea nitrogen; CA^++^, calcium; PHOS, phosphorus; CR, creatinine; GLU, glucose; Na^+^, sodium; K^+^, potassium; TP, total protein
