# Supplementary material for: Growth Responses of Preterm Pigs Fed Formulas with Different Protein Levels and Supplemented with Leucine or β-Hydroxyl β-Methylbutyrate
Source: Nutrients. 2018 May 18;10(5):636. doi: 10.3390/nu10050636 (PMC5986515; doi:10.3390/nu10050636)
Supplement: Supplementary file 1 [file nutrients-10-00636-s001.zip › Nutrients-293566 Suppl Table 3.docx]

Supplemental Table S3. Blood chemistries for Experiment 2 pigs at the start (day 0) of being fed the high protein formula with alanine (HP+Ala), leucine (HP+Leu), and β-hydroxyl β-methylbutyrate (HP+HMB) and every day thereafter until necropsy. Values are means and standard errors.

| DAY & GROUP | ALP^1^  (U/L) | ALT (U/L) | AMY (U/L) | TBIL (mg/dL) | BUN (mg/dL) | CA++ (mg/dL) | PHOS (mg/dL) | | CRE (mg/dL) | GLU (mg/dL) | NA+ | K+ | TP (g/dL) |
| --- | --- | --- | --- | --- | --- | --- | --- | --- | --- | --- | --- | --- | --- |
| 0 | 925+68 | 19+1 | 421+29 | 0.36+0.02 | 17.6+1.11 | 11.6+0.3 | 4.6+0.3 | | 0.52+0.02 | 175+32 | 138+1 | 5.6+0.2 | 2.4+0.1 |
| 1 HP-Ala | 886+87 | 18+1 | 515+ | 0.37+0.03 | 12.0+0.9 | 10.8+0.3 | 4.1+0.3 | | 0.47+0.03 | 68+6 | 139+1 | 5.5+0.1 | 2.5+.1 |
| HP-Leu | 618+69 | 17+1 | 398+48 | 0.30+0.03 | 16.1+2.5 | 9.8+0.7 | 4.2+0.4 | 0.49+0.04 | | 79+5 | 140+1 | 5.7+0.2 | 2.6+0.1 |
| HP-HMB | 876+150 | 20+2 | 474+68 | 0.38+0.02 | 17.2+2.3 | 9.6+0.9 | 3.7+0.2 | | 0.42+0.05 | 59+6 | 139+1 | 5.7+0.5 | 2.4+0.1 |
| 2 HP-Ala | 1027+110 | 28+8 | 583+61 | 0.32+0.01 | 9.3+1.4 | 9.8+0.4 | 4.7+0.2 | | 0.41+0.02 | 71+5 | 139+1 | 5.2+0.1 | 2.7+0.1 |
| HP-Leu | 867+57 | 25+4 | 545+53 | 0.30+0.00 | 10.6+1.6 | 9.5+0.4 | 5.0+0.4 | | 0.46+0.03 | 69+6 | 139+1 | 5.0+0.2 | 2.7+0.3 |
| HP-HMB | 1492+110 | 19+1 | 577+30 | 0.38+0.05 | 15.5+2.5 | 9.7+2.3 | 5.5+1.9 | | 0.43+0.02 | 74+10 | 140+0 | 5.7+0.1 | 2.6+0.1 |
| 3 HP-Ala | 986+121 | 21+2 | 599+49 | 0.27+0.01 | 20.5+1.8 | 9.9+0.3 | 5.8+0.3 | | 0.41+0.03 | 62+3 | 139+0 | 5.4+0.1 | 3.1+0.0 |
| HP-Leu | 882+126 | 18+1 | 557+42 | 0.31+0.01 | 18.9+1.9 | 10.3+0.6 | 5.2+0.4 | | 0.38+0.03 | 62+5 | 139+1 | 5.2+0.2 | 3.0+0.1 |
| HP-HMB | 1519+235 | 26+1 | 486+16 | 0.28+0.02 | 28.3+3.9 | 11.7+0.2 | 4.7+0.3 | | 0.33+0.03 | 64+6 | 142+1 | 6.6+0.2 | 3.3+0.1 |
| 4 HP-Ala | 748+97 | 19+1 | 586+82 | 0.27+0.02 | 25.7+2.0 | 9.8+0.2 | 6.5+0.2 | | 0.41+0.04 | 57+4 | 138+1 | 5.3+0.2 | 3.5+0.1 |
| HP-Leu | 619+62 | 21+2 | 485+58 | 0.25+0.02 | 25.5+4.4 | 9.1+0.6 | 6.1+0.4 | | 0.39+0.03 | 54+4 | 137+1 | 5.5+0.2 | 3.4+0.1 |
| HP-HMB | No data | | | | | | | | | | | | |
| 5 HP-Ala | 747+90 | 20+1 | 571+63 | 0.28+0.02 | 27.8+1.5 | 9.8+0.4 | 5.6+0.3 | | 0.37+0.02 | 58+4 | 138+1 | 5.1+0.1 | 3.5+0.1 |
| HP-Leu | 711+75 | 19+1 | 524+39 | 0.23+0.02 | 29.9+1.6 | 10.4+0.3 | 6.2+0.3 | | 0.40+0.03 | 62+4 | 136+1 | 5.3+0.1 | 3.7+0.1 |
| HP-HMB | 1435+303 | 25+2 | 499+15 | 0.27+0.03 | 40.3+6.9 | 12.0+0.2 | 6.3+0.6 | | 0.37+0.03 | 69+3 | 138+1 | 5.7+0.2 | 3.4+0.1 |
| 6/7 HP-Ala | 550+43 | 20+1 | 424+57 | 0.23+0.02 | 34.8+3.0 | 10.5+0.5 | 6.2+0.3 | | 0.37+0.04 | 72+7 | 136+1 | 5.1+0.3 | 3.7+0.2 |
| HP-Leu | 654+94 | 21+1 | 521+35 | 0.24+0.02 | 32.1+3.0 | 10.6+0.3 | 6.1+0.3 | | 0.48+0.02 | 72+8 | 138+1 | 5.4+0.2 | 3.7+0.2 |
| HP-HMB | 676+184 | 24+1 | 466+112 | 0.27+0.03 | 41.3+12.7 | 10.9+1.6 | 4.7+0.7 | | 0.47+0.12 | 98+30 | 138+1 | 5.3+0.9 | 3.4+0.8 |

^1^ 1 ALP, alkaline phosphatase; ALT, alanine aminotransferase, AMY, amylase; TBIL, total bilirubin; BUN, blood urea nitrogen; CA++, calcium; PHOS, phosphorus; CR, creatinine; GLU, glucose; Na+, sodium; K+, potassium; TP, total protein
