# Supplementary material for: Growth Responses of Preterm Pigs Fed Formulas with Different Protein Levels and Supplemented with Leucine or β-Hydroxyl β-Methylbutyrate
Source: Nutrients. 2018 May 18;10(5):636. doi: 10.3390/nu10050636 (PMC5986515; doi:10.3390/nu10050636)
Supplement: Supplementary file 1 [file nutrients-10-00636-s001.zip › Nutrients-293566 Suppl File 1.docx]

**Ingredients used to prepare the low and high protein formulas.**

The dry formulas were prepared by Animix (Juneau, WI) to provide when fed (per L) either 100 g or 50 g of protein, 50 g lactose as the sole source of carbohydrate, and 47.5 g fat. Milk protein isolate (60% caseinate and 40% whey) was used as the protein source and was provided by Abbott Nutrition. The fat was provided by Animix using a 7/60 dry fat blend that provides at least 80% fat and 7% protein with the fat component consisting of 80% animal fat (lard) and 20% coconut oil preserved with BHA, and including dried whey, casein, and lecithin. The MCT oil was added at the time the dry powder was mixed with warm water. The multivitamin source and multitrace element additive (Baxter Healthcare Corporation) provided (% based on reported composition) anhydrous dicalcium phosphate (85.13), Ferrous sulfate, 20% (2.598), Bioplex zinc 15% (2.462), Sel-Plex 2700 (Alltech) as source of selenium (2.278), taurine (1.56), vitamin E 50% (1.225), vitamin C (1.02), zinc sulfate (1.008), carnitine 50% (0.6117), bioplex copper 10 (0.5606), biotin 2.0% (0.4575), vitamin A 500 (0.2857), niacinamide (0.2222), 25 D-Ca pantothenate (0.1464), Bioplex manganese 15% (0.1213), vitamin K 33% (0.0618), riboflavin (0.0611), vitamin D3 500 (0.0571), manganese sulfate (0.0488), vitamin B12 1% (0.0260), pyridoxine hydrochloride (0.0245), thiamine monohydrate (0.0183), calcium iodate (0.0159), folic acid (0.0037). The LEC/STAR487/MO B is an emulsifier consisting of a blend of polyoxyethylene glycol (400 mono and dioleate), lecithin, and mineral oil (Animix, Juneau, WI).
