# Supplementary material for: Growth Responses of Preterm Pigs Fed Formulas with Different Protein Levels and Supplemented with Leucine or β-Hydroxyl β-Methylbutyrate
Source: Nutrients. 2018 May 18;10(5):636. doi: 10.3390/nu10050636 (PMC5986515; doi:10.3390/nu10050636)
Supplement: Supplementary file 1 [file nutrients-10-00636-s001.zip › Nutrients-293566 Suppl Table 1.docx]

Supplemental Table S1. Hematology values at the end of parenteral nutrition (0) for all pigs and at necropsy (means and SEM) for preterm pigs from Experiment 1 fed the formulas with high protein (HP) or low protein with alanine (LP+Ala) or leucine (LP+Leu) and pigs from Experiment 2 fed the high protein formula with either alanine (HP+Ala), leucine (HP+Leu), or HMB (HP+HMB). Values are means and standard errors.

|  | | Experiment 1 | | | Experiment 2 | | |
| --- | --- | --- | --- | --- | --- | --- | --- |
|  | 0 | HP | LP+Ala | LP+Leu | HP+Ala | HP+Leu | HP+HMB |
| WBC^1^ (10^9^/L) | 3.1+0.2 | 17.5+1.6 | 15.9+2.0 | 19.3+1.8 | 21.2+2.4 | 16.4+3.1 | 18.6+2.4 |
| LYM (10^9^/L) | 2.8+0.2 | 14.6+2.1 | 12.8+2.5 | 17.4+2.7 | 20.4+2.8 | 16.2+3.1 | 18.1+ 2.4 |
| MON (10^9^/L) | 0.2+0.01 | 0.3+0.1 | 0.3+0.1 | 0.1+0.0 | 0.1+0.01 | 0.1+0.05 | 0.09+0.01 |
| GRA (10^9^/L) | 0.1+0.1 | 2.7+1.4 | 2.8+1.6 | 1.8+1.2 | 0.8+0.7 | 0.06+0.03 | 0.4+0.3 |
| RBC (10^12^/L) | 4.3+0.1 | 4.2+0.3 | 4.6+0.3 | 4.3+0.2 | 4.7+0.2 | 4.4+.02 | 5.0+0.4 |
| HGB (g/dL) | 8.3+0.2 | 7.2+0.5 | 7.9+0.5 | 7.3+0.3 | 7.9+0.4 | 7.3+0.4 | 8.9+0.7 |
| HCT | 27.0+0.6 | 23.0+1.3 | 24.5+1.4 | 22.9+0.9 | 25.1+1.1 | 23.8+1.1 | 28.3+2.2 |
| MCV | 63+0.5 | 55+1 | 54+1.3 | 53.3+0.6 | 54+1 | 54+1 | 57+0.8 |
| MCH (mg) | 19.4+0.3 | 17.1+0.3 | 17.1+0.5 | 16.9+0.2 | 16.9+0.3 | 16.6+0.4 | 18.1+0.3 |
| PLT (10^9^/L) | 224+12 | 384+63 | 560+87 | 527+53 | 392+101 | 336+36 | 307+57 |

^1^ WBC, white blood cells; LYM, lymphocytes; MON, monocytes; GRA, granulocytes; RBC, red blood cells; HGB, hemoglobin; HCT, hematocrit; MCV, mean cell volume; MCH, mean cell hemoglobin; PLT, platelets
